# Emotion Regulation, Parasympathetic Function, and Psychological Well-Being

**DOI:** 10.3389/fpsyg.2022.879166

**Published:** 2022-08-03

**Authors:** Ryan L. Brown, Michelle A. Chen, Jensine Paoletti, Eva E. Dicker, E. Lydia Wu-Chung, Angie S. LeRoy, Marzieh Majd, Robert Suchting, Julian F. Thayer, Christopher P. Fagundes

**Affiliations:** ^1^Department of Psychological Sciences, Rice University, Houston, TX, United States; ^2^Department of Psychiatry and Behavioral Sciences, McGovern Medical School at UTHealth, Houston, TX, United States; ^3^Department of Psychological Science, University of California, Irvine, Irvine, CA, United States; ^4^Department of Behavioral Sciences, The University of Texas MD Anderson Cancer Center, Houston, TX, United States; ^5^Department of Psychiatry and Behavioral Sciences, Baylor College of Medicine, Houston, TX, United States

**Keywords:** emotion regulation, heart rate variability (HRV), resilience, depression, perceived stress

## Abstract

The negative emotions generated following stressful life events can increase one’s risk of depressive symptoms and promote higher levels of perceived stress. The process model of emotion regulation can help distinguish between adaptive and maladaptive emotion regulation strategies to determine who may be at the greatest risk of worse psychological health across the lifespan. Heart rate variability (HRV) may affect these relationships as it indexes aspects of self-regulation, including emotion and behavioral regulation, that enable an individual to dynamically adapt to the changing demands of both internal and external environments. In this study, we expected individual differences in resting vagally mediated HRV to moderate the influence of emotion regulatory strategies among our sample of 267 adults. We found support for the hypothesis that higher vagally mediated HRV buffers against the typical adverse effects of expressive suppression when evaluating depressive symptoms and found weak support when considering perceived stress. There was no evidence for an interaction between cognitive reappraisal and vagally mediated HRV but there was a significant, negative association between cognitive reappraisal and depressive symptoms and perceived stress. Future work may determine if intervening on either emotion regulation strategies or HRV may change these within-persons over time.

## Introduction

Stressful life events and their accompanying negative emotions can increase the risk of depressive symptoms, major depressive disorder, and higher levels of perceived stress. Interpersonal stresses, or stress from major interpersonal losses such as death and divorce, are robust predictors of major depressive disorder (Slavich and Irwin, 2014). Even in the context of other interpersonal stresses, spousal bereavement is often considered one of the most stressful possible life events, as widowed spouses have significantly elevated depressive symptoms and perceived stress (Holmes and Rahe, 1967; Stroebe et al., 2007; Fagundes and Wu, 2020). During the first year of bereavement, about half of all widowed spouses will meet the criteria for major depressive disorder (Clayton, 1990; Zisook and Shuchter, 1991; Stroebe et al., 2007). Typically, older adults experience major depressive disorder at lower rates than younger adults, with 15% of community-dwelling older adults meeting the criteria for clinically significant depressive symptoms but not major depressive disorder (Blazer, 2003; Fiske et al., 2009). Compared to younger adults, older adults are better at emotional regulation and less reactive to stress, two important protective factors for depression (Garnefski and Kraaij, 2006; Neupert et al., 2007; Fiske et al., 2009).

Emotion regulation skills play an important role in the relationship between stressful life events and mental health outcomes, such as depression. Using the process model of emotion regulation to study the effects of cognitive reappraisal and expressive suppression can help distinguish between adaptive and maladaptive emotion regulation strategies when facing a stressful life event (Gross, 1998; Gross and John, 2003). Specifically, cognitive reappraisal occurs when an individual alters the meaning of the situation by changing how one thinks about the situation, whereas expressive suppression involves directly changing the experiential, behavioral, or physiological components of an emotional response after response tendencies have begun, such as a facial expression (Gross, 1998). Cognitive reappraisal typically presents more adaptive responses than expressive suppression, but the effects can vary by context (Gross, 1998; Gross and John, 2003). Broadly, previous work indicates that cognitive reappraisal is associated with decreased depressive symptoms and can buffer the relationship between stress and depressive symptoms (Troy et al., 2010; Andreotti et al., 2013), while expressive suppression is associated with increased depressive symptoms, less positive emotion, more negative emotion, feelings of inauthenticity, and sympathetic nervous system responses (Gross and Levenson, 1993; Harris, 2001; Gross and John, 2003; Demaree et al., 2006; Moore et al., 2008; Nezlek and Kuppens, 2008). The discrepancy between these strategies can be traced to differences in the timing of implementation and cognitive effort. Cognitive reappraisal is an *antecedent-focused* strategy and is implemented before an experienced emotion has initiated changes in behavior and peripheral physiological responses, while expressive suppression, a *response-focused* strategy, is only implemented after these changes are already underway (Gross and John, 2003). Expressive suppression may also be more cognitively taxing than cognitive reappraisal (Richards and Gross, 2000), which affects an individual’s ability to perform other essential executive functions related to psychological and physiological outcomes during stress, such as increased levels of rumination and experiential avoidance (Moore et al., 2008).

Individual differences in vagally mediated heart rate variability (HRV), a marker of parasympathetic nervous system functioning measured by an individual’s beat-to-beat variability in heart rate, may help explain who has lower levels of stress and depressive symptoms (Thayer and Lane, 2000; Thayer et al., 2009; Fagundes et al., 2018). Importantly, HRV can serve as an index of an individual’s self-regulatory abilities and physiological responses to stressful life events (Porges et al., 1994; Bridgett et al., 2013; Carnevali et al., 2018). Differences in HRV can be used to understand risk and resilience patterns that can impact how individuals adapt to stress (Fagundes et al., 2012; Sbarra and Borelli, 2013). Dynamically adapting to changing environmental demands and stressors is a core component of resilience processes (Kalisch et al., 2017). Those with high HRV may respond more flexibly under stress, while individuals with low HRV may experience adverse outcomes when coping and adapting to stress (Thayer et al., 2021). For example, when discussing an ongoing conflict with one’s partner, men generally experienced more negative affect the more they were suppressing their emotional expression; however, for men with higher vagally mediated HRV, there was no association between suppression and negative affect (Geisler and Schröder-Abé, 2015).

Based on these findings, we expected individual differences in resting vagally mediated HRV to moderate the influence of emotion regulatory strategies in our sample. We hypothesized that higher reported frequencies of expressive suppression would be associated with elevated depressive symptoms (Hypothesis 1a) and perceived stress (Hypothesis 2a) and that these associations with depressive symptoms and perceived stress would be buffered by high vagally mediated HRV (Hypothesis 1b and Hypothesis 2b, respectively). That is, we expected better self-regulation (i.e., high vagally mediated HRV) to be a resilience factor. Conversely, we hypothesized that higher reported frequencies of cognitive reappraisal would be associated with lower depressive symptoms (Hypothesis 3a) and perceived stress (Hypothesis 4a) and that those who reported more frequently using cognitive reappraisal and had high vagally mediated HRV would have the lowest levels of depressive symptoms (Hypothesis 3b) and perceived stress (Hypothesis 4b).

## Materials and Methods

### Participants

As part of a larger longitudinal-observational study investigating the biological mechanisms underlying greater cardiovascular disease risk during bereavement, our sample includes 267 participants measured across three study visits. The 267 total participants included 167 spousally bereaved participants and 100 control participants (*M* = 68 years; 68% women; see Table 1 for all sample characteristics). Bereaved participants met inclusion criteria if they had experienced the death of their spouse within 3 months of their first visit, if they had been married to their spouse for at least 3 years, and if they were able to read and write in English. Control participants were age-, sex-, and education-matched to their bereaved counterparts as part of the larger study. Control participants were not excluded based on their relationship status, but they must not have experienced the death of their spouse within the last 5 years. All participants were able to read and write in English. Participants were not included in the study if they had visual or auditory impairments that interfered with their ability to read or hear questionnaires, were pregnant or nursing, had an autoimmune disease, had been divorced in the last year, had experienced the death of a loved one in the last year (excluding spouse for those in the bereaved group), or if they were undergoing cancer treatment. The first visit was approximately 3 months after the death of the bereaved participants’ spouses, the second visit was approximately 4 months after the death of their spouse, and the third visit was approximately 6 months after the death of the spouse; the control participants’ visits were identically spaced.

**TABLE 1:** Means, standard deviations, and correlations with confidence intervals for key study variables.

Variable	*M*	SD	1	2	3	4	5	6	7	8
1. Depressive symptoms*^a^*	12.86	10.34								
2. Perceived stress*^a^*	11.83	6.86	0.76** [0.73, 0.79]							
3. RMSSD*^b^*	26.54	25.35	−0.09* [−0.16, −0.02]	−0.08* [−0.15, −0.00]						
4. Expressive suppression*^b^*	13.80	4.95	0.11** [0.04, 0.18]	0.05 [−0.02, 0.12]	−0.01 [−0.08, 0.07]					
5. Cognitive reappraisal*^b^*	31.46	6.52	−0.10** [−0.17, −0.03]	−0.10** [−0.17, −0.03]	−0.10** [−0.17, −0.03]	0.02 [−0.05, 0.09]				
6. Age*^b^*	68.51	9.82	−0.15** [−0.22, −0.08]	−0.22** [−0.29, −0.15]	0.15** [0.08, 0.22]	0.07* [0.00, 0.14]	−0.04 [−0.11, 0.03]			
7. BMI*^b^*	28.28	5.86	0.11** [0.04, 0.19]	0.10** [0.02, 0.17]	−0.01 [−0.09, 0.06]	−0.06 [−0.13, 0.01]	−0.02 [−0.10, 0.05]	−0.07 [−0.14, 0.01]		
8. Comorbid conditions*^b^*	0.56	2.70	−0.07 [−0.14, 0.00]	−0.08* [−0.15, −0.01]	−0.00 [−0.08, 0.07]	0.05 [−0.02, 0.12]	0.01 [−0.06, 0.08]	0.01 [−0.07, 0.08]	−0.00 [−0.07, 0.07]	
9. Education*^b,c^*	0.80	1.11	0.03 [−0.04, 0.10]	−0.04 [−0.11, 0.03]	−0.01 [−0.08, 0.06]	0.10** [0.03, 0.17]	0.03 [−0.04, 0.11]	0.02 [−0.05, 0.09]	0.23** [0.16, 0.30]	−0.03 [−0.10, 0.04]

*M and SD are used to represent mean and standard deviation, respectively. Values in square brackets indicate the 95% confidence interval for each correlation. The confidence interval is a plausible range of population correlations that could have caused the sample correlation (Cumming, 2014).*

*^a^Assessed at Visits 1, 2, and 3.*

*^b^Assessed at Visit 1 only.*

^c^Education was assessed using an ordinal scale, ranging from 0 (graduate/professional training) and 5 (less than 7 years of schooling). *p < 0.05; **p < 0.01.

### Measures

#### Depressive Symptoms

Participants completed the 20-item Center for Epidemiologic Studies Depression Scale (CES-D; Radloff, 1977) at each visit. The CES-D is a widely utilized measure of depressive symptoms that asks participants to describe the way they have felt in the prior week (e.g., “I felt sad”). Higher scores on this scale indicate greater depressive symptomatology, although the scale can also be used with a cut-score to indicate a clinically significant level of depressive symptoms (Radloff, 1977). Depressive symptoms were measured as a continuous variable by reverse-coding as needed and summing the items, which resulted in good reliability (α_*V*1_ = 0.91, α_*V*2_ = 0.91, and α_*V*3_ = 0.91).

#### Perceived Stress

Participants completed the 10-item Perceived Stress Scale (PSS; Cohen et al., 1983) at each visit. The PSS asks participants to describe how often they have felt stressed in the last month on a scale of 0 (never) to 4 (very often). An example item reads, “How often have you been upset because of something that happened unexpectedly?” The items were reverse-coded as needed and summed; the scale resulted in good reliability (α_*V*1_ = 0.90, α_*V*2_ = 0.89, and α_*V*3_ = 0.90).

#### Emotion Regulation

At the baseline visit, participants completed the 10-item Emotion Regulation Questionnaire (ERQ; Gross and John, 2003). The ERQ assesses the frequency of participants’ emotional regulation *via* reappraisal and expressive suppression on a 7-point Likert scale of 1 (Strongly disagree) to 7 (Strongly agree). The ERQ yields two separate summed scores for cognitive reappraisal (e.g., “I control my emotions by changing the way I think about the situation I’m in”) and expressive suppression (e.g., “I keep my emotions to myself”). Scale items were reverse coded as needed and summed; the resulting cognitive reappraisal scale had good reliability (α_*V*1_ = 0.82) and expressive suppression scale had fair reliability (α_*V*1_ = 0.69).

#### Heart Rate Variability

Heart rate variability was measured for 5 continuous minutes at the baseline visit with the Polar s810 wristwatch and the Polar H10 heart rate sensor. The 1,000 Hz sampling rate provides valid and reliable ECG data non-invasively (Müller et al., 2019). All procedures followed the recommendations of the Task Force of the European Society of Cardiology and the North American Society of Pacing Electrophysiology (Malik et al., 1996). To prepare HRV data for analysis, we used the KUBIOS HRV Premium analysis software to process the raw interbeat intervals for artifact removal (Tarvainen et al., 2009). Following artifact correction in KUBIOS, each segment was hand-scored to ensure no ectopic beats or other artifacts were present. KUBIOS provides values for vagally mediated (parasympathetic) HRV using the square root of mean successive differences (RMSSD); higher values indicate higher vagally mediated HRV. RMSSD is determined by calculating the differences between consecutive interbeat (RR) intervals before squaring, adding, and averaging the values. Finally, the square root of the values are used as the RMSSD (Stein et al., 1994; Malik, 1996). RMSSD is highly correlated with spectral derived measures of HRV, but is preferred to spectral indices as it is less affected by respiration and other artifacts than spectral indices of HRV (Penttila et al., 2001).

#### Comorbid Conditions

At the baseline visit, participants completed the 19-item Charlson Comorbidity Index (CCI; Charlson et al., 1994). The CCI is the most common measure of comorbidity. The 19 items are assigned a weight based on their likelihood of influencing 1-year mortality. The CCI was used as a covariate in all adjusted analyses.

#### Other Covariates

Demographic factors, including bereavement status, age, gender, education, and body mass index (BMI) were included as covariates in all adjusted models. All covariates were assessed at the first visit time point. Bereavement status was dummy coded as 0 (control) or 1 (bereaved). Age and gender were both captured *via* self-report. Education was assessed using an ordinal scale, ranging from 0 (graduate/professional training) and 5 (less than 7 years of schooling). Finally, BMI was computed from participant height and weight as measured during their first visit in units of kg/m^2^.

### Analytic Method

Preliminary statistical analysis included assessment of normality of distributions and examination for skewness and kurtosis. To satisfy the assumption of normality of residuals we used a square root transformation for depressive symptoms for all analyses involving depressive symptoms. Generalized linear mixed modeling (GLMM), a multilevel regression analytic technique, was used to fit the outcome variables (i.e., depressive symptoms and perceived stress) as a function of the interaction between log RMSSD and emotion regulation strategies (i.e., expressive suppression and cognitive reappraisal), controlling for constituent main effects. Only depressive symptoms and perceived stress were time-varying. The person was the upper level and time was the lower level in all multilevel analyses. We fit each model as an adjusted model with the same set of fixed covariates (months since baseline visit, bereavement status, age, gender, comorbidities, BMI, and education). When we test one component of emotion regulation, we also include the other component of emotion regulation as a covariate (e.g., cognitive reappraisal is a covariate in models focusing on the interaction between expressive suppression and RMSSD). Age, comorbidities, log RMSSD, months since baseline visit, frequency of expressive suppression, and frequency of cognitive reappraisal were centered for analyses. We used a median imputation for missing data on BMI (1.2% of the variable’s total values) and education (0.8% of the variable’s total values).

For each outcome (depressive symptoms; perceived stress), a model comparison approach was used to determine the functional form of change over time (i.e., testing linear, quadratic, and higher-order polynomial effects) and the optimal random effects structure (i.e., evaluating the need to include random intercept and/or slope terms). The Akaike information criterion (AIC) was used to determine the best-fitting model for each outcome. We chose the model based on a significance test between these models and after identifying the lowest AIC value, which indicates the best, most parsimonious fit with the least information lost relative to other models (Bozdogan, 1987; Vrieze, 2012). In each case, the best-fitting model included a random intercept. Further, models for each primary outcome variable demonstrated non-linear change over time, and model comparison indicated that including a linear and a quadratic effect best captured the functional form of these changes. Models including only a linear effect or including higher-order effects (e.g., cubic) effects demonstrated inferior fit.

Apart from reliability analyses conducted in SPSS (IBM Corp, 2016), all analyses were conducted in the R statistical computing environment (R Core Team, 2019). Multilevel analyses used the package *nlme* (Pinheiro et al., 2020), used *ggplot2* (Wickham, 2016) and *ggeffects* (Lüdecke et al., 2021) for visualization, and *apaTables* to generate tables (Stanley, 2018).

## Results

Descriptive statistics and correlations between key variables can be found in Table 1.

### Emotion Regulation, Heart Rate Variability, and Depressive Symptoms

#### Bereavement Status and Depressive Symptoms

Unsurprisingly, participants in the study who were bereaved (vs. control) reported higher levels of depressive symptoms across the study, *b* = 1.20, 95% CI [0.88, 1.52], *p* < 0.001. Although our bereaved participants understandably reported higher depressive symptoms than our control participants, on average, depressive symptoms within both the control group (*M* = 8.21, SD = 7.77) and the bereaved group (*M* = 15.60, SD = 10.70) remained below the cut-score (16 or greater; Radloff, 1977) that indicates risk for clinical depression across the 6-month study period. Accordingly, neither the bereaved nor control group would be classified as at risk for clinical depression.

There was neither a reliable interaction between bereavement status and expressive suppression associated with depressive symptoms, *b* = −0.01, 95% CI [−0.07, 0.05], *p* = 0.73, nor bereavement status and cognitive reappraisal associated with depressive symptoms, *b* = −0.00, 95% CI [−0.05, 0.04], *p* = 0.33, nor bereavement status and log RMSSD associated with depressive symptoms, *b* = −0.17, 95% CI [−0.59, 0.25], *p* = 0.44.

### Emotion Regulation and Depressive Symptoms

We found support for Hypothesis 1a as there was a reliable, positive association between expressive suppression and depressive symptoms in adjusted models, *b* = 0.05, 95% CI [0.01, 0.75], *p* = 0.005, such that those who reported a higher frequency of expressive suppression had elevated depressive symptoms throughout the study compared to those who reported lower frequency of expressive suppression. In these models, we also found support for Hypothesis 3a as there was a reliable, negative association between cognitive reappraisal and depressive symptoms, *b* = −0.03, 95% CI [−0.05, −0.003], *p* = 0.031, such that those who reported a greater frequency of cognitive reappraisal had lower depressive symptoms throughout the study.

**FIGURE 1:**
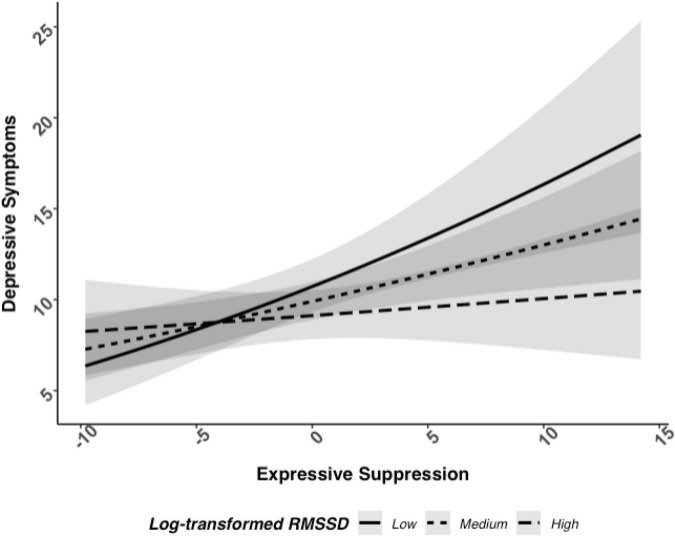
Relationship between log RMSSD, expressive suppression, and depressive symptoms when adjusted with covariates.

We analyzed the potential interaction between log RMSSD and frequency of expressive suppression related to depressive symptoms. Here, there was evidence of an interaction between log RMSSD and frequency of expressive suppression associated with depressive symptoms, *b* = −0.04, 95% CI [−0.08, −0.00], *p* = 0.042 (see Figure 1; and Table 2 for full model results). Next, we examined simple slope tests and determined that individuals with low HRV (i.e., −1 SD) had a positive relationship between expressive suppression and depressive symptoms (*b* = 0.08, *p* < 0.001), but individuals with high HRV (i.e., +1 SD) did not have a significant relationship between expressive suppression and depressive symptoms (*b* = 0.02, *p* = 0.48). These findings support Hypothesis 1b and indicate that higher HRV may reduce or eliminate the association between expressive suppression and depressive symptoms.

**TABLE 2:** Frequency of expressive suppression and RMSSD associated with depressive symptoms over the course of 6 months.

	Depressive symptoms			Depressive symptoms			Depressive symptoms		
	(unadjusted model)			(adjusted for covariates)			(sensitivity analysis with medications)		
			
Predictors	Estimates	CI	*p*	Estimates	CI	*p*	Estimates	CI	*p*
(Intercept)	2.40	2.14 to 2.67	**<0.001**	1.06	0.23 to 1.90	**0.012**	1.07	0.25 to 1.89	**0.010**
RMSSD* Expressive suppression	–0.05	−0.09 to −0.00	**0.032**	–0.04	−0.08 to −0.00	**0.042**	–0.04	−0.08 to 0.00	0.051
Months since baseline	–0.13	−0.16 to −0.09	**<0.001**	–0.13	−0.16 to −0.09	**<0.001**	–0.12	−0.16 to −0.09	**<0.001**
Months since baseline ^2	0.04	0.01 to 0.06	**0.004**	0.04	0.01 to 0.06	**0.003**	0.04	0.01 to 0.06	**0.004**
Bereavement status	1.19	0.87 to 1.50	**<0.001**	1.22	0.91 to 1.53	**<0.001**	1.15	0.84 to 1.45	**<0.001**
Cognitive reappraisal	–0.02	−0.05 to −0.00	**0.048**	–0.02	−0.05 to −0.00	**0.030**	–0.02	−0.05 to −0.00	**0.045**
RMSSD	–0.22	−0.43 to −0.01	**0.040**	–0.17	−0.38 to 0.03	0.096	–0.11	−0.31 to 0.09	0.282
Expressive suppression	0.03	0.00 to 0.06	**0.035**	0.05	0.02 to 0.08	**0.003**	0.04	0.01 to 0.07	**0.005**
Education				–0.02	−0.16 to 0.12	0.769	–0.03	−0.16 to 0.11	0.677
Age				–0.02	−0.04 to −0.01	**0.003**	–0.02	−0.04 to −0.01	**0.001**
Gender				0.38	0.06 to 0.71	**0.020**	0.30	−0.02 to 0.63	0.067
BMI				0.04	0.01 to 0.06	**0.005**	0.04	0.01 to 0.06	**0.008**
Comorbid conditions				–0.01	−0.07 to 0.04	0.632	–0.01	−0.07 to 0.04	0.647
Beta blockers							0.07	−0.31 to 0.45	0.711
Antidepressants							0.63	0.26 to 0.99	**0.001**
**Random effects**									
σ^2^	0.57			0.57			0.57		
τ_00_	1.38 _*id*_			1.28 _*id*_			1.21 _*id*_		
ICC	0.71			0.69			0.68		
*N*	267 _*id*_			267 _*id*_			264 _*id*_		
Observations	743			743			734		
Marginal *R*^2^/conditional *R*^2^	0.198/0.766			0.249/0.769			0.271/0.766		

*^2^Indicates the squared, quadratic effect of time.*

*Bold values indicate significant effects.*

The symbol “*” indicates the interaction between these two variables.

In a sensitivity analysis to test the robustness of this effect among the participants (*n* = 264) who provided information on their medication use, the interaction between log RMSSD and frequency of expressive suppression associated with depressive symptoms was essentially the same after controlling for antidepressant and beta-blocker use (*b* = −0.04, 95% CI [−0.08, 0.00], *p* = 0.051).

There was no evidence for an interaction between cognitive reappraisal and log RMSSD associated with depressive symptoms, *b* = −0.00, 95% CI [−0.03, 0.03], *p* = 0.90. Thus, Hypothesis 3b was not supported.

### Emotion Regulation, Heart Rate Variability, and Perceived Stress

#### Bereavement Status and Perceived Stress

Participants in the study who were bereaved (vs. control) reported higher levels of perceived stress across the study, *b* = 2.33, 95% CI [0.76, 3.90], *p* = 0.004. There was neither a reliable interaction between bereavement status and expressive suppression associated with perceived stress, *b* = 0.12, 95% CI [−0.18, 0.41], *p* = 0.44, nor bereavement status and cognitive reappraisal associated with perceived stress, *b* = 0.02, 95% CI [−0.20, 0.24], *p* = 0.88, nor bereavement status and log RMSSD associated with perceived stress, *b* = −1.08, 95% CI [−3.08, 0.91], *p* = 0.30.

### Emotion Regulation and Perceived Stress

There was support for Hypothesis 2a based on an association between expressive suppression and perceived stress, *b* = 0.17, 95% CI [0.29, 0.32], *p* = 0.021; those who reported a greater frequency of expressive suppression had higher perceived stress throughout the study. There was also support for Hypothesis 4a in these models as we identified an association between cognitive reappraisal and perceived stress, *b* = −0.12, 95% CI [−0.23, −0.02], *p* = 0.027; those who reported a greater frequency of cognitive reappraisal had lower perceived stress throughout the study.

**FIGURE 2:**
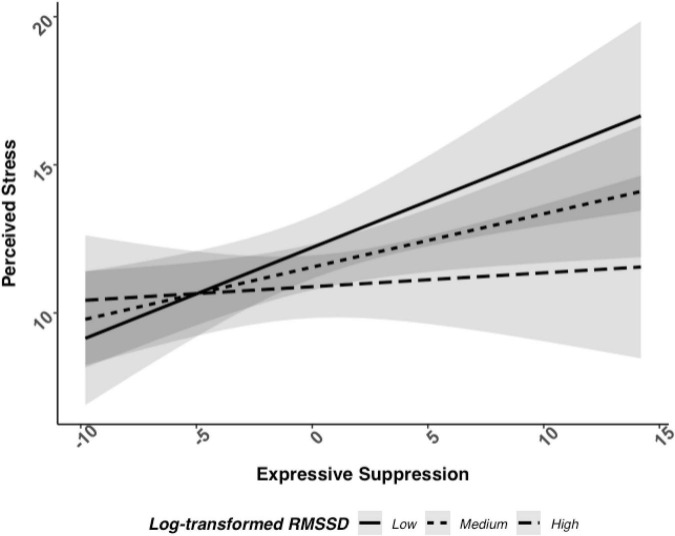
Relationship between log RMSSD, expressive suppression, and perceived stress when adjusted with covariates.

Next, we analyzed perceived stress to determine if there was an interaction between log RMSSD and frequency of expressive suppression. There was only weak evidence of an interaction between log RMSSD and frequency of expressive suppression associated with perceived stress, which did not reach statistical significance in adjusted models, *b* = −0.18, 95% CI [−0.38, 0.01], *p* = 0.069 (see Figure 2; and Table 3 for full model results). The level of statistical significance did not change when controlling for antidepressant and beta-blocker use. Simple slope tests followed the same pattern as depressive symptoms: individuals with low HRV (i.e., −1 SD) had a positive relationship between expressive suppression and perceived stress (*b* = 0.31, *p* = 0.004), while individuals with high HRV (i.e., +1 SD) did not have a significant relationship between expressive suppression and perceived stress (*b* = 0.05, *p* = 0.64). Thus, Hypothesis 2b was only partially supported.

**TABLE 3:** Frequency of expressive suppression and RMSSD associated with perceived stress over the course of 6 months.

	Perceived stress			Perceived stress			Perceived stress		
	(unadjusted model)			(adjusted for covariates)			(sensitivity analysis with medications)		
			
Predictors	Estimates	CI	*p*	Estimates	CI	*p*	Estimates	CI	*p*
(Intercept)	10.15	8.86 to 11.44	**<0.001**	4.39	0.40 to 8.38	**0.031**	4.38	0.50 to 8.26	**0.027**
RMSSD * Expressive suppression	–0.21	−0.41 to 0.00	0.050	−0.18	−0.38 to 0.01	0.069	−0.17	−0.36 to 0.02	0.078
Months since baseline	–0.50	−0.64 to −0.36	**<0.001**	−0.50	−0.64 to −0.36	**<0.001**	−0.49	−0.63 to −0.35	**<0.001**
Months since baseline ^2	0.12	0.02 to 0.23	**0.020**	0.13	0.02 to 0.23	**0.017**	0.13	0.02 to 0.23	**0.017**
Bereavement status	2.26	0.70 to 3.82	**0.005**	2.37	0.88 to 3.86	**0.002**	2.05	0.59 to 3.51	**0.006**
Cognitive reappraisal	–0.11	−0.23 to −0.00	**0.049**	−0.12	−0.23 to −0.02	**0.024**	−0.11	−0.22 to −0.00	**0.041**
RMSSD	–1.16	−2.20 to −0.12	**0.029**	−0.91	−1.89 to 0.08	0.071	−0.55	−1.52 to 0.41	0.260
Expressive suppression	0.08	−0.07 to 0.23	0.284	0.18	0.03 to 0.33	**0.016**	0.17	0.02 to 0.31	**0.025**
Education				−0.56	−1.22 to 0.09	0.092	−0.56	−1.20 to 0.08	0.086
Age				−0.14	−0.21 to −0.07	**<0.001**	−0.14	−0.22 to −0.07	**<0.001**
Gender				2.92	1.36 to 4.48	**<0.001**	2.39	0.84 to 3.93	**0.002**
BMI				0.15	0.02 to 0.27	**0.023**	0.14	0.02 to 0.26	**0.027**
Comorbid conditions				−0.18	−0.45 to 0.08	0.177	−0.18	−0.43 to 0.08	0.174
Beta blockers							−0.40	−2.19 to 1.39	0.660
Antidepressants							3.62	1.87 to 5.37	**<0.001**
**Random effects**									
σ^2^	10.40			10.40			10.39		
τ_00_	34.85 _*id*_			30.52 _*id*_			28.35 _*id*_		
ICC	0.77			0.75			0.73		
*N*	267 _*id*_			267 _*id*_			264 _*id*_		
Observations	743			743			734		
Marginal R^2^/conditional R^2^	0.079/0.788			0.176/0.791			0.215/0.789		

*^2^Indicates the squared, quadratic effect of time.*

*Bold values indicate significant effects.*

The symbol “*” indicates the interaction between these two variables.

Next, we determined that there was not a significant interaction between cognitive reappraisal and log RMSSD associated with perceived stress, *b* = −0.11, 95% CI [−0.26, 0.03], *p* = 0.14. Thus, Hypothesis 4b was not supported.

### Ancillary Analyses

Because this sample is part of a larger study identifying what puts bereaved spouses at greater risk for cardiovascular disease, we also examined whether any of these effects differed based on the bereavement status of the participant in ancillary analyses. Specifically, we examined whether the key interactions of interest may differ based on whether participants were bereaved or not. If there were differences, this could reflect a sensitive period for widow(er)s if higher vagally mediated HRV was not protective, but we made no specific hypotheses regarding group differences. We explored whether there may be a three-way interaction associated with depressive symptoms between bereavement status, emotion regulation strategies, and log RMSSD in ancillary analyses.

There was neither evidence for the interaction between bereavement status, expressive suppression, and log RMSSD, *b* = −0.35, 95% CI [−0.92, 0.22], *p* = 0.25, nor for the interaction between bereavement status, cognitive reappraisal, and log RMSSD, *b* = −0.35, 95% CI [−0.79, 0.10], *p* = 0.13, associated with depressive symptoms.

We also examined whether there may be a three-way interaction associated with perceived stress between bereavement status, emotion regulation strategies, and log RMSSD in ancillary analyses. Again, there was neither evidence for the interaction between bereavement status, expressive suppression, and log RMSSD, *b* = −0.15, 95% CI [−0.56, 0.25], *p* = 0.47, nor for the interaction between bereavement status, cognitive reappraisal, and log RMSSD, *b* = −0.06, 95% CI [−0.37, 0.25], *p* = 0.69, associated with perceived stress.

## Discussion

Participants who reported using expressive suppression with greater frequency and had higher vagally mediated HRV at rest had lower depressive symptoms severity than participants who also used expressive suppression with greater frequency but had lower vagally mediated HRV. There was also weak evidence for this association between expressive suppression and vagally mediated HRV associated with perceived stress in a similar pattern as predicted depressive symptoms, but this was not statistically significant. These effects did not vary based on whether participants were bereaved or controls, suggesting that higher vagally mediated HRV may buffer against the harmful psychological effects of expressive suppression for adults generally and following a highly stressful life event like spousal bereavement. Broadly, participants who reported using cognitive reappraisal with greater frequency reported lower depressive symptoms and perceived stress levels. However, there was no evidence for an interaction between cognitive reappraisal and vagally mediated HRV associated with depressive symptoms or perceived stress.

Generally, we found support for the notion that higher vagally mediated HRV is a resilience factor that protects against the typical adverse effects of expressive suppression. In our sample, vagally mediated HRV was protective when examining depressive symptoms and followed a similar pattern, though the interaction overall was not significant, for perceived stress. These results are consistent with findings from other research groups that higher vagally mediated HRV reduces or eliminates effects of expressive suppression on negative affect during conflict (Geisler and Schröder-Abé, 2015), enables people to spontaneously suppress negative facial expressions in an adaptive and socially appropriate manner when exposed to a negative film clip (Pu et al., 2010), and protects against depressive symptoms for those with emotion regulation difficulties (Fantini-Hauwel et al., 2020). Although meta-analytic results indicate only a small correlation between self-regulation and HRV (Holzman and Bridgett, 2017), these results may be interpreted as an interplay between this bio-marker of one’s self-regulatory ability and the frequency participants report using expressive suppression. It will be important to determine if these relationships can be modified within-persons; future work may examine whether interventions seeking to increase vagally mediated HRV also successfully reduce the impact of expressive suppression on psychological well-being.

As our sample primarily consists of older adults, our findings indicate the relationship between emotion regulation strategies and psychological well-being in older adulthood. Positive emotional experiences are more important to older adults than younger adults (Carstensen et al., 2003) and older adults may be more likely to engage in emotion regulation strategies other than cognitive reappraisal and expressive suppression (e.g., situation selection; Urry and Gross, 2010). Despite the potentially low base rate of older adults using cognitive reappraisal and expressive suppression, we found that cognitive reappraisal is inversely related to perceived stress and depressive symptoms. This finding aligns with other work showing that positive reappraisal is negatively associated with depressive symptoms and may be especially effective for older adults (Garnefski and Kraaij, 2006; Shiota and Levenson, 2009). Older adults and younger adults are equally able to implement expressive suppression (Kunzmann et al., 2005; Shiota and Levenson, 2009), although this strategy was directly related to increased perceived stress and depressive symptoms in our results. In addition, we found that expressive suppression and HRV interacted such that people with low HRV experienced higher depressive symptoms when they employed more expressive suppression, while people with high HRV had no such experience. Broadly, these findings are situated in the context of our sample as older adults are less likely to utilize expression suppression than younger adults (John and Gross, 2004; Kunzmann et al., 2005; Phillips et al., 2008).

### Limitations and Future Directions

Despite the strengths of this study, there are some limitations that could be avenues for future research. The present findings are correlational; hence, causality cannot be established by the current data. Identifying which variable (expressive suppression or HRV) is truly the moderator of this relationship is another limitation of this study – more fine-grained longitudinal analyses of changes in expressive suppression frequency and/or HRV over time would benefit the development of interventions. Although we consider HRV to be an important resilience factor, we used a single baseline measurement of HRV as an indication of how flexibly one can respond to the changing demands of their environment, which is limiting because it does not capture the process of adapting over time in response to a specific stressor (Kalisch et al., 2017). We also did not assess HPA axis activity or fronto-amygdala circuitry that may be involved in these relationships. Because expressive suppression and cognitive reappraisal are associated with different neural networks and differing levels of amygdala attenuation (Vanderhasselt et al., 2013; Dörfel et al., 2014; Lopez et al., 2018), future work may examine how variation in amygdala-frontal connectivity during emotion regulation impacts changes in daily cortisol levels (Urry et al., 2006; Hakamata et al., 2017). Assessing these parameters would provide a more holistic view of the relative influence of each of these systems on depressive symptoms and perceived stress.

Additionally, the operationalization of emotion regulation has some limitations. The ERQ currently measures self-reported cognitive reappraisal and expressive suppression *frequency*, not actual efficacy in implementation. This study assumes that self-reported cognitive reappraisal frequency is a proxy for emotion regulation ability. This assumption can be made because those who implement cognitive reappraisal more frequently have shown to be better at cognitive reappraisal since they are naturally incorporating it more into their daily lives (Gross and John, 2003). Because emotion regulation tactics can be implemented automatically and unconsciously, there are limitations in assessing frequency in a self-report measure. Future experimental studies will help address these limitations by providing information on emotion regulation ability and strategy frequency. Further, the ERQ only measures the frequency of two emotion regulation strategies, but other strategies as categorized by Gross (1998) may also promote positive health outcomes (Sheppes et al., 2011). Cognitive reappraisal has also been defined by two different tactics of reinterpretation and distancing, which have been differentially effective given the intensity of the perceived stress (McRae et al., 2012). Future work should explore these same relationships with other emotion regulation strategies and tactics longitudinally. These relationships were measured in older adulthood and may not generalize to other developmental stages. Finally, the present study included a largely non-Hispanic White sample; thus, future studies should investigate these associations in more diverse samples.

The present study found evidence that high HRV buffers the negative effects of expressive suppression on depressive symptoms and perceived stress over 6 months. Thus, vagal tone, a proxy for trait-level self-regulation, may help reduce the impact of more frequently using less adaptive emotion regulation strategies. While correlational, the present results support HRV as a candidate biological mechanism through which individuals regulate their depressive symptoms and perceived stress, aligning with prior findings (Segerstrom and Nes, 2007; Thayer et al., 2012). Because early-life stress may alter these relationships, future work may examine neurodevelopment, vagally mediated HRV, and emotion regulation longitudinally throughout childhood and emerging adulthood. These results motivate future longitudinal, experimental work to determine if receiving training to improve HRV may be causally related to changes in stress and depressive symptoms during bereavement or for non-bereaved adults. For example, interventions such as vagus nerve stimulation or cognitive behavioral therapy (Carney et al., 2000; Bonaz et al., 2021) may help alleviate the influence of greater expressive suppression frequency on psychological well-being by increasing one’s vagally mediated HRV. Broadly, this finding aligns with a previous meta-analysis of 24 studies that showed HRV biofeedback training is associated with improvements in stress and anxiety (Goessl et al., 2017). Increased knowledge of potentially adaptive regulatory options may help people prepare to cope with current and future stressful circumstances.

## Data Availability Statement

The raw data supporting the conclusions of this article will be made available by the authors, without undue reservation.

## Ethics Statement

The studies involving human participants were reviewed and approved by the Rice University, Institutional Review Board. The patients/participants provided their written informed consent to participate in this study.

## Author Contributions

CF and JT developed the parent study concept and study design. CF oversaw all aspects of data collection and manuscript preparation. JT oversaw the heart rate variability analysis for the parent study, which includes the data presented here. RB and MC began the investigation for this specific manuscript and performed the data analysis and interpretation under the supervision of CF. RS aided in data analysis and interpretation throughout the revision process. RB, MC, JP, and ED drafted and edited the manuscript. EW-C, AL, MM, JT, RS, and CF provided critical revisions. All authors approved the final version of the manuscript for submission.

## Conflict of Interest

The authors declare that the research was conducted in the absence of any commercial or financial relationships that could be construed as a potential conflict of interest.

## Publisher’s Note

All claims expressed in this article are solely those of the authors and do not necessarily represent those of their affiliated organizations, or those of the publisher, the editors and the reviewers. Any product that may be evaluated in this article, or claim that may be made by its manufacturer, is not guaranteed or endorsed by the publisher.
